# Multi-Location External Workload Profile in Women’s Basketball Players. A Case Study at the Semiprofessional-Level

**DOI:** 10.3390/s21134277

**Published:** 2021-06-22

**Authors:** Carlos D. Gómez-Carmona, David Mancha-Triguero, José Pino-Ortega, Sergio J. Ibáñez

**Affiliations:** 1 Research Group in Optimization of Training and Sports Performance (GOERD), Sport Science Faculty, University of Extremadura, Av. de la Universidad s/n, 10005 Caceres, Spain; davidmancha@unex.es (D.M.-T.); sibanez@unex.es (S.J.I.); 2 BioVetMed & Sport Sci Research Group, Physical Activity and Sports Department, Sport Science Faculty, University of Murcia, Argentina Street s/n, San Javier, 30720 Murcia, Spain

**Keywords:** microtechnology, impacts, human body, accelerometry, inertial devices

## Abstract

The external workload measured in one anatomical location does not determine the total load supported by the human body. Therefore, the purpose of the present study was to characterize the multi-location external workload through PlayerLoad_RT_ of 13 semi-professional women’s basketball players, as well as to analyze differences among anatomical locations (inter-scapulae line, lumbar region, 2× knee, 2× ankle) and laterality (left vs. right) during five tests that represent the most common movements in basketball—(a) linear locomotion, 30-15 IFT; (b) acceleration and deceleration, 16.25-m RSA (c) curvilinear locomotion, 6.75-m arc (d) jump, Abalakov test (e) small-sided game, 10’ 3 vs. 3 10 × 15-m. Statistical analysis was composed of a repeated-measures t-test and eta partial squared effect size. Regarding laterality, differences were found only in curvilinear locomotion, with a higher workload in the outer leg (*p* < 0.01; *η_p_*^2^ = 0.33–0.63). In the vertical profile, differences among anatomical locations were found in all tests (*p* < 0.01; *η_p_*^2^ = 0.56–0.98). The nearer location to ground contact showed higher values except between the scapulae and lumbar region during jumps (*p* = 0.83; *η_p_*^2^ = 0.00). In conclusion, the multi-location assessment of external workload through a previously validated test battery will make it possible to understand the individual effect of external workload in each anatomical location that depends on the type of locomotion. These results should be considered when designing specific strategies for training and injury prevention.

## 1. Introduction

Basketball can be considered one of the most popular sports in the world because of the large number of people involved, and its dynamic characteristics [1]. In Spain, basketball is the second sport with the most licenses and the first in this category in women [2]. Regarding physical and physiological requirements, basketball involves intermittent efforts that combine high-intensity actions with low-intensity periods. Women’s basketball players covered 4–6 km per game [3], performed 400–550 changes of direction [4], supported 450–650 a.u PlayerLoad^TM^ (PL_RT_ = 45–55 a.u.) [5] and performed 10–20 high-intensity accelerations (>3 m/s^2^) [6]. The external workload influenced the internal workload with 3–5 mmoL of blood lactate and 82–90% HR_MAX_ [3,7]. So, the comprehension of internal and external workload and its individualization could be important for managing load in basketball [8].

For external workload assessment, time-motion analysis (TMA) through video cameras or radiofrequency technologies in indoor conditions (e.g., ultra-wideband) has been utilized to analyze the volume and intensity of players’ locomotion on the court [8]. To complete the data provided by TMA, the use of accelerometers has increased exponentially. These sensors provide the changes in acceleration in the three planes of movement performed by the players as a result of the interaction with gravitational forces and teammates/opponents [9]. One of the injury risk factors is the accumulated external workload detected through accelerometry-based indexes, identifying a direct effect with injury risk throughout the season in Australian football [10], soccer [11], or rugby [12]. The main drawback of these studies is that they evaluated the external workload in one anatomical location (scapulae) which seems to be insufficient due to multi-joint complexity during sports movements [9]. 

Through the assessment of external workload in different locations simultaneously, a higher workload has been found in the lower limbs in comparison with the upper limbs during laboratory and field tests [13,14]. In basketball, the most commonly injured area has been in the lower limbs, with lateral ankle sprains and knee pathologies especially relevant in female players [15]. Therefore, to understand how the external workload is assimilated by the musculoskeletal structures of the human body, multi-location assessment is a useful alternative [9], and a field test battery has been designed recently with this aim [16]. Therefore, the purposes of the present study were to—(a) characterize the multi-location external workload profile in semi-professional women’s basketball players, and (b) to analyze the differences in external workload regarding anatomical locations (scapulae, lumbar region, knee, ankle) and laterality (left vs. right) during the most common movements in basketball.

## 2. Materials and Methods

### 2.1. Design

A cross-sectional design was employed to characterize the multi-location external workload profile of women’s basketball players during the most common movements in basketball through a previously validated field test battery [16] in the following order—(a) curvilinear locomotion, 6.75-m arc test; (b) jump capacity, Abalakov test; (c) acceleration and deceleration profile, 16.25-m RSA test; (d) linear locomotion, 30-15 IFT test; and (e) game conditions, 10’ 3 vs. 3 small-sided game. To assess the multi-location external workload profile, PlayerLoad_RT_ was recorded using six WIMUPRO^TM^ inertial devices that were placed in six anatomical locations—(1) inter-scapulae line, (2) lumbar region, (3) right knee, (4) left knee, (5) right ankle, and (6) left ankle. In the present study, an ecological treatment was given as all measures were taken during the tests without intervention.

### 2.2. Participants

Thirteen semi-professional women’s basketball players participated voluntarily in the present study. The anthropometric characteristics per playing position were shown in Table 1. All the players took part in the reserve team of an elite-level women’s basketball team in Spain (Liga Femenina 1, first division of women’s basketball). All the participants met the following inclusion criteria—(a) over two-months experience of high-level monitoring during training and competition, and (b) absence of musculoskeletal injury or health problems [17].

The club managers, technical staff and players were previously informed about the investigation details and signed informed consent forms. The informed consent of under-18 players was subscribed additionally by their legal guardians. The study was performed based on the ethical guidelines of the Declaration of Helsinki (2013) and approved by the Bioethics Committee of the University of Extremadura (registration number 232/2019). 

### 2.3. Procedures

The study lasted 3 weeks with four sessions. During the first week, the anthropometrical assessment (height, weight, and body composition), the explanation of the study purposes and the collection of the informed consent forms were carried out. The anthropometrical assessment was performed using an 8-electrode segmental monitor MC-780MA model (TANITA, Tokyo, Japan) and a rod stadiometer (SECA, Hamburg, Germany).

In the second week, two familiarization sessions with the protocol and multi-location monitoring were performed. Finally, in the fourth session, the assessment of the external workload profile was made using a previously validated field battery [16] composed of five tests—(a) 6.75-m arc test to evaluate the capacity for curvilinear locomotion at maximum speed, where five repetitions were performed in each direction with a 1-min rest between repetitions and a 5-min rest between directions (5× left and 5× right); (b) the Abalakov test to evaluate the jump capacity with arm coordination, where five repetitions were performed with a 30-s rest between repetitions; (c) 16.25-m RSA test to evaluate the acceleration and deceleration phase (from the free-throw line to the 6.75-m line, with 5 m of deceleration between the 6.75-m line and the basket), where five repetitions were performed with a 1-min between-repetition rest; (d) 30-15 IFT to evaluate the aerobic capacity and the linear locomotion on the court, where during the test, fractions of 30-s running was combined with fractions of 15-s rest; and (e) a 3 vs. 3 10 × 15-m small-sided game to evaluate the physical fitness during real-game conditions during 10 min with an official referee and rules. The rest time was active at low intensity in all cases to ensure optimal recovery between repetitions and between tests [1].

To assess the multi-location external workload during the field test battery, Player Load by the RealTrack Systems company (PL_RT_, accelerometer-derived measurement of total body load in its 3 axes—vertical, anterior-posterior and medial-lateral) [9] was recorded through WIMU PRO^TM^ inertial measurement units (RealTrack Systems, Almeria, Spain). Each device was composed of four 3D accelerometers (full-scale range—±16, ±16, ±32 and ±400 g) to improve the accuracy and reliability through the fusion of data from the accelerometers based on the redundancy principle [18]. Also, other sensors are incorporated in the device (three 3D gyroscopes, a 3D magnetometer, a 10-Hz GPS, a 20-Hz UWB). The accelerometers presented very high between-device reliability in static (CV = 0.23–0.78%; Bias = 0.00–0.02 g) and dynamic conditions in different anatomical locations (scapulae, lower back, knee and ankle) (CV = 2.05–2.96%; Bias = 0.00–0.04 g) [19], and PL_RT_ presented satisfactory reliability (ICC = 0.96–0.99; CV = 4.65–6.54%) and convergent validity results (HR_AVG_, *r* > 0.99; SmO_2_, *r* < −0.69) to quantify neuromuscular load [20]. During recording, the sampling frequency of the microsensors was set at 100 Hz. 

The time selection during tests in each player on the timeline of the WIMU PRO^TM^ inertial devices was carried out in real-time during the assessment through three hardware devices—(a) Windows tablet with SVIVO^TM^ software, (b) Ant+ pushbuttons and, (c) photocells with Ant+ pushbuttons. These hardware devices present nearly perfect accuracy and reliability [21].

**Figure 1:**
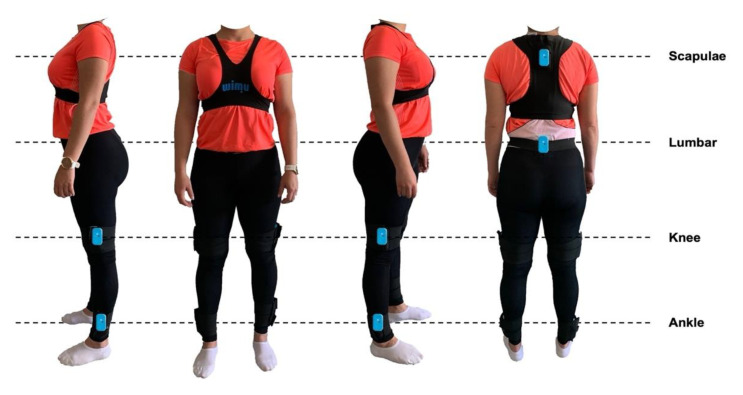
Placement of the inertial devices in women’s basketball players.

Previous to placing the inertial devices on the players, they were calibrated following the manufacturer’s recommendations to ensure the perfect functioning of the microelectromechanical sensors [19]. Players were cited 30 min before the testing to locate the six inertial devices in six anatomical locations simultaneously through a specific one-piece sports vest—(i) back (inter-scapulae line), (ii) lumbar region (L3-L5, center of mass), (iii) knee (3-cm above the kneecap’s crack) and (iv) ankle (3-cm above the lateral malleolus) [19]. In the knees and ankles, the devices were on the external side (see Figure 1 for more details). Then, a specific warm-up was performed to achieve the best physical performance of the players 20 min before the start of the testing. The distribution of the warm-up was—(1) 10 min of moderate activity, (2) 5 min of dynamic stretching, and (3) 3 min of light activity to prepare for the start of the testing. Between tests, a 5-min active recovery period was carried out.

### 2.4. Statistical Analysis

First, the data were downloaded from the six inertial devices. The software SPRO^TM^ was used to sync the data on the same timeline to be able to compare the recorded data during the same joint action and to calculate and export PL_RT_ data. Then, an exploratory analysis to determine the distribution and the homogeneity of data was performed using the Shapiro–Wilk test and Levene test, respectively, showing a parametric distribution. 

A descriptive analysis (mean and standard deviation, M ± SD) was performed. To compare data among anatomical locations both in all players and per player, a repeated-measures t-test was used. The effect sizes were obtained by eta partial squared (*η_p_*^2^) and were interpreted as—*η_p_*^2^ < 0.01 trivial, *η_p_*^2^ = 0.01–0.06 low, *η_p_*^2^ = 0.06–0.14 moderate, and *η_p_*^2^ > 0.14 high [22]. The significance level was established at *p* < 0.05. Data analysis was performed with the Statistical Package for the Social Sciences (SPSS Statistics, version 24, IBM Corporation, Armonk, NY, USA) and figures were designed with GraphPad Prism (GraphPad Ltd., version 8, La Jolla, CA, USA). Figures represent a scatter dot plot with mean (black line), whiskers (standard deviation) and color dots (basketball players).

## 3. Results

### 3.1. Multi-Location External Workload Profile

**Figure 2:**
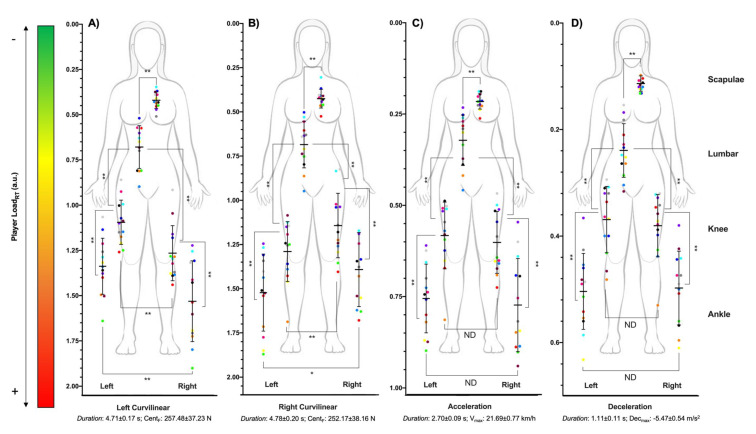
Multi-location external workload profile of semi-professional women’s basketball players in curvilinear locomotion ((**A**) left and (**B**) right direction) and speed changes ((**C**) acceleration and (**D**) deceleration). ** Statistical differences (*p* < 0.01); * Statistical differences (*p* < 0.05); ND: No statistical differences.

The multi-location external workload profile of women’s basketball players is shown during curvilinear locomotion in Figure 2A,B and change of speed in Figure 2C,D. The PL_RT_ supported during curvilinear locomotion was in the scapulae (left: 0.42 ± 0.05; right: 0.43 ± 0.05), lumbar region (left: 0.68 ± 0.12; right: 0.69 ± 0.13), right knee (left: 1.27 ± 0.15; right: 1.14 ± 0.18), left knee (left: 1.10 ± 0.12; right: 1.29 ± 0.17), right ankle (left: 1.53 ± 0.22; right: 1.39 ± 0.21) and left ankle (left: 1.34 ± 0.16; right: 1.52 ± 0.22). On the other hand, The PL_RT_ supported during changes of speed was in the scapulae (acceleration, acc: 0.22 ± 0.02; deceleration, dec: 0.11 ± 0.01), lumbar region (acc: 0.32 ± 0.07; dec: 0.24 ± 0.05), right knee (acc: 0.60 ± 0.08; dec: 0.38 ± 0.06), left knee (acc: 0.58 ± 0.09; dec: 0.37 ± 0.06), right ankle (acc: 0.77 ± 0.13; dec: 0.50 ± 0.07) and left ankle (acc: 0.76 ± 0.09; dec: 0.50 ± 0.07).

**Figure 3:**
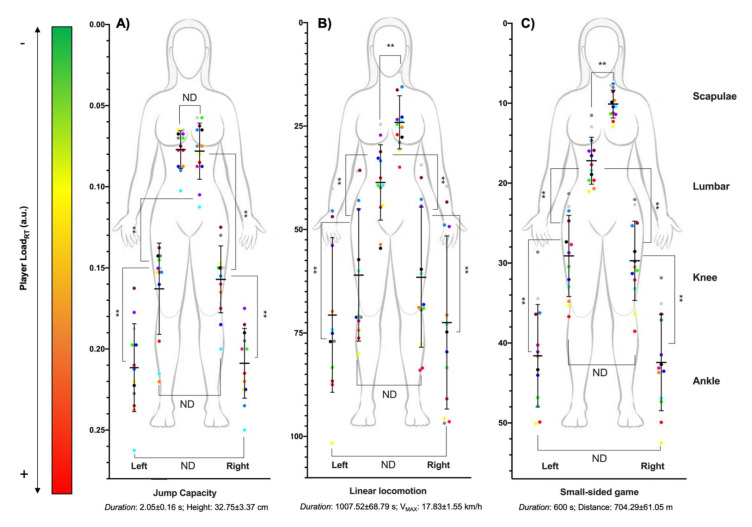
Multi-location external workload profile of semi-professional women’s basketball players in (**A**) jumps, (**B**) linear locomotion and (**C**) small-sided games. ** Statistical differences (*p* < 0.01); ND: No statistical differences.

Figure 3 shows the multi-location external workload profile of women’s basketball players during jumps (Figure 3A), linear locomotion (Figure 3B) and small-sided games (Figure 3C). The PL_RT_ supported during jumps was in the scapulae (0.08 ± 0.02), lumbar region (0.08 ± 0.01), right knee (0.16 ± 0.02), left knee (0.16 ± 0.03), right ankle (0.21 ± 0.02) and left ankle (0.21 ± 0.03); during linear locomotion it was in the scapulae (24.08 ± 6.42), lumbar region (38.63 ± 9.11), right knee (61.52 ± 16.92), left knee (60.99 ± 15.97), right ankle (72.48 ± 20.91) and left ankle (70.67 ± 18.68); and during small-sided games it was in the scapulae (10.11 ± 1.74), lumbar region (17.20 ± 2.94), right knee (29.71 ± 4.80), left knee (29.11 ± 4.09), right ankle (42.44 ± 6.04) and left ankle (41.60 ± 6.41).

### 3.2. Vertical and Horizontal Differences

The vertical and horizontal differences in external workload suffered by the players in the different anatomical locations were shown in Table 2. In the vertical profile, differences were found among all anatomical locations with higher values in the location nearer to ground contact (left curvilinear: *p <* 0.01, *t =* 4.47–13.60, *η_p_*^2^ = 0.62–0.94; right curvilinear: *p <* 0.01, *t =* 3.87–13.30, *η_p_*^2^ = 0.56–0.94; acceleration: *p <* 0.01, *t =* 5.08–11.44, *η_p_*^2^ = 0.68–0.92; deceleration: *p <* 0.01, *t* = 5.31–10.55, *η_p_*^2^ = 0.70–0.90; jump: *p <* 0.01, *t =* 8.48–19.98, *η_p_*^2^ = 0.86–0.97; linear: *p <* 0.01, *t =* 4.76–7.73, *η_p_*^2^ = 0.65–0.83; small-sided game: *p <* 0.01, *t =* 12.91–23.39, *η_p_*^2^ = 0.93–0.98), except between the scapulae and lumbar region during jumps (*p* = 0.83, *t =* 0.22, *η_p_*^2^ = 0.00).

In the horizontal profile, differences were found in curvilinear locomotion with higher external workload in the outer leg in comparison with the inner leg in the knee (left direction: *p* < 0.01, *t =* 4.53, *η_p_*^2^ = 0.63; right direction: *p <* 0.01, *t =* 3.05, *η_p_*^2^ = 0.44) and ankle (left direction: *p* < 0.01, *t =* 4.92, *η_p_*^2^ = 0.56; right direction: *p <* 0.01, *t =* 2.16, *η_p_*^2^ = 0.33), except during left curvilinear locomotion in player 5 with a higher workload in the left ankle, and during right curvilinear locomotion in players 6 and 13 with higher values in the right knee, and players 3 and 9 with higher values in the right ankle. However, no differences between knees and ankles were found during the acceleration (*p >* 0.31; *t =* 0.91–0.97; *η_p_*^2^ < 0.01) and deceleration phase (*p <* 0.01; *t =* 0.43–0.95; *η_p_*^2^ < 0.01), jumps (*p >* 0.31; *t =* 0.64–1.06; *η_p_*^2^ < 0.01), linear locomotion (*p >* 0.12; *t =* 0.43–1.68; *η_p_*^2^ < 0.01) or small-sided games (*p >* 0.14; *t =* 0.16–1.56; *η_p_*^2^ < 0.01).

## 4. Discussion

The assessment of external workload is widely extended in men’s basketball, but the evidence in women’s basketball is scant [3]. Thanks to the use of inertial devices, tracking sensors and microtechnology (accelerometers) have been integrated into the same device to explain how the player moves on the court (positioning) and how these movements affect the load (impacts) supported [9]. Because of this dual measurement, it has been recommended to locate the device on the interscapular line for better reception of the positioning signal [23]. However, the question is whether recording at a single anatomical location sufficient to determine the load supported by the different musculoskeletal structures of the body? Nedergaard et al. [13] determined that accelerometers only detect the load on the location or segment to which they are attached. In this respect, the traditional evaluation of the load at a single anatomical point seems to be insufficient, and it seems necessary to evaluate different body locations simultaneously and to achieve more accurate load quantification [14,16]. Therefore, the present study aimed to make a first approach to the characterization of the multi-location profile of external workload in semiprofessional women’s basketball players, as well as to evaluate the differences among anatomical locations in the vertical (scapulae, lumbar region, knee and ankle) and horizontal profile (left vs. right knee and ankle).

From the results obtained, a global vision of the multi-location external workload profile in the most common movements in basketball in semi-professional women’s basketball players has been identified. The volume and intensity of locomotion influenced the behavior of external workload, as well as their type and direction [9,19]. This is because the propulsive and braking forces against the ground have a direct effect on the accelerometry-based workload [24]. The greatest differences between the scapulae-lumbar region were found in the deceleration phase, between the lumbar region-knee in jumps and between the knee-ankle during small-sided games. On the other hand, the smallest differences between the scapulae-lumbar region were found in the jumps, between the lumbar region-knee in the deceleration phase and between the knee-ankle in linear locomotion. These specific workload profiles should be considered for training design, where core strength and stability [25], unilateral and bilateral strength for lower limb musculoskeletal structures [26] and running gait programs [27] seem to be useful to improve the distribution of the external workload among anatomical locations.

Regarding the horizontal profile, differences were found between legs in curvilinear locomotion while differences were not found in the rest of the locomotion (acceleration, deceleration, jumps, linear and small-sided games). The gait biomechanics during curvilinear locomotion differ from linear locomotion because the outer and inner leg present different functions [28]. This causes an imbalance between the force exerted by each leg, which should be trained according to specific considerations such as balance, body control, and core strength and stability [25,29,30]. In addition, as knee and ankle injuries are produced commonly without contact and during locomotion or actions that imply a change of direction (e.g., cutting, pivoting, blocking out), the use of high-intensity curvilinear locomotion seems to be fundamental for training and performance assessment, with the 6.75-m arc test being a valid tool for these two purposes [16,31].

Anthropometric characteristics and physical and physiological capabilities have a direct influence on the internal and external load supported by players during training and competition [32]. In the present study, high variability was found in the multi-location external workload profile of women’s basketball players, especially in the lower limbs (knee and ankle), where the higher standard deviations were obtained. In this regard, the identification of individual profiles may be fundamental for identifying the reference profile after an injury as well as for analyzing the evolution of physical fitness throughout a season [33,34]. 

In addition, the highest external workload differences were found between the lumbar region and knee in the women’s basketball players evaluated. Women’s basketball players have the highest musculoskeletal absorption in this segment and a large intensity of impact supported by the ankle which reaches the knee due to anthropometric (wider hips, higher Q angle, higher tibiofemoral angle and genu recurvatum) and physical factors (lower center of mass and lateral displacement of it away from the knee joint, greater mean anterior pelvic tilt, hip anteversion and torso rotation) [4,35]. These aspects affect the trunk and hip flexion angles as well as hip adduction and internal rotation during sports movements, making women players prone to lower limb injuries [36,37]. So, the trunk, hip and knee joints need to be considered in women’s basketball players due to the high rate of anterior cruciate ligament injuries and the effect of external variables such as fatigue and the menstrual cycle on this injury process [38,39]. Therefore, the injury prevention protocols, training tasks and recovery programs should focus on the lower limbs in comparison with the upper limbs. 

While the results of this investigation have provided the first approach to multi-location external workload assessment of women’s basketball players, with six inertial devices on the different upper (scapulae and lumbar region) and lower limb (knee and ankle) locations to characterize vertical absorption and the differences related to laterality, some limitations to the study must be acknowledged. Firstly, the data obtained cannot be extrapolated to other populations with different individual characteristics because the sample studied is reduced and specific (13 women’s basketball players at the semiprofessional level). Another limitation concerns the validity and reliability of the inertial devices. Although these devices are widely used by sports scientists and professional teams in different modalities of individual and team sports, and the reliability and validity of WIMU PRO^TM^ inertial devices [19,20] have been proved in different conditions (laboratory vs. field, static vs. dynamic) and anatomical locations (inter-scapulae line, lumbar region, knees and ankles), the criterion measures are not considered as gold standard methods. For this reason, the obtained results should be taken with caution and future research could assess the validity and reliability of this device with respect to gold standard methods to provide more consistency to the obtained findings.

Finally, the multi-location external workload assessment through a validated field test battery [16] has proven its usefulness to identify individual profiles. In this respect, future research could evaluate the external workload through this assessment protocol during training and competition to detect the specific workload supported by each body location and design specific training programs for performance improvement or injury prevention. If this assessment was performed in a large population, the identification of injury profiles based on the difference between anatomical locations in the vertical profile (absorption by musculoskeletal structures) and horizontal profile (laterality) could be achieved.

## 5. Conclusions

Through the previously validated field test battery, the present study shows a first approach to the multi-location external workload profile of women’s basketball players during the most common movements in basketball. Women’s basketball players supported the higher external workload in the lower limbs (ankle and knee) that is related to the distance to the ground contact. The greatest difference between anatomical locations was found in the knee-lumbar segment (42.53% ± 5.78%) while the smallest difference was found in the knee-ankle segment (21.48% ± 5.56%). Besides, high variability was found among players due to the large amplitude in the standard deviation, especially in the lower limb.

Regarding locomotion, a specific profile was found. The highest between-location differences were found in the scapulae-lumbar region during decelerations, in the lumbar region-knee during jumps and in the knee-ankle during the small-sided game. No differences were found only in the scapulae-lumbar segment during jumps. On the other hand, differences in lateral profile were found between curvilinear and linear locomotion with higher impacts in the outer leg in comparison to the inner leg regardless of the curvilinear locomotion direction.

From these conclusions, different practical applications could be mentioned to improve the training for women’s basketball teams—(a) the recovery protocols in the lower limb should be more in-depth in comparison to the upper limb because the greatest load is supported by the knee and ankle, also the greatest differences between locations were found in the knee-lumbar segment (musculoskeletal activity of thigh and gluteus); (b) the low absorption in the knee-ankle segment may be associated with the higher risk of knee injuries in female players (e.g., anterior cruciate ligament) so that an improvement in musculoskeletal absorption in this segment (calf, tibia and soleus) together with gait programs would be recommended to reduce the injury risk; (c) because each type of locomotion presented a specific multi-location external workload profile, the identification of these profiles will help in the design of specific training programs; (d) curvilinear locomotion presented a higher external workload in the outer knee and ankle so the training tasks should consider the different motor patterns of each leg for the improvement of players’ performance and injury prevention. 

## Figures and Tables

**Table 1:** Anthropometric characteristics of women’s basketball players in the present study.

	Guard (*n* = 3)	Forward (*n* = 5)	Center (*n* = 5)	Total (*n* = 13)
Age (years)	17.33 ± 0.58	17.81 ± 2.66	20.32 ± 3.57	18.49 ± 2.27
Height (m)	1.65 ± 0.05	1.70 ± 0.05	1.81 ± 0.06	1.73 ± 0.08
Weight (kg)	59.33 ± 8.13	64.26 ± 9.38	72.66 ± 11.46	66.64 ± 10.94
BMI (kg/m^2^)	21.80 ± 3.87	22.30 ± 3.26	22.41 ± 2.96	22.25 ± 3.15
Fat mass (%)	23.60 ± 7.86	26.29 ± 3.97	28.31 ± 2.80	26.72 ± 4.68
Muscle mass (%)	72.56 ± 7.52	69.98 ± 3.77	68.05 ± 2.68	69.58 ± 4.47

**Table 2:** Differences in vertical and horizontal external workload profile in the most common movements in basketball.

Test	Statistics	Vertical Differences					Horizontal Differences	
		Scapulae ^1^ vs. Lumbar ^2^	Lumbar ^1^ vs. Right Knee ^2^	Lumbar ^1^ vs. Left Knee ^2^	Right Knee ^1^ vs. Right Ankle ^2^	Left Knee ^1^ vs. Left Ankle ^2^	Right ^1^ vs. Left ^2^ Knee	Right ^1^ vs. Left ^2^ Ankle
Left curvilinear	*t* *(p)*	8.38 (<0.01)	13.60 (<0.01)	10.97 (<0.01)	4.47 (<0.01)	7.58 (<0.01)	4.53 (<0.01)	4.92 (<0.01)
	*η_p_^2^*, *ES*	0.85 *high*	0.94 *high*	0.91 *high*	0.62 *high*	0.83 *high*	0.63 *high*	0.56 *high*
	*%_diff_,* *1-e-2*	38.06 0-0-13	46.11 0-0-13	37.76 0-0-13	17.38 0-0-13	18.11 0-0-13	13.40 12-1-0	12.65 11-1-1
Right curvilinear	*t* *(p)*	7.35 (<0.01)	10.41 (<0.01)	13.15 (<0.01)	13.30 (<0.01)	3.87 (<0.01)	3.05 (<0.01)	2.16 (0.04)
	*η_p_*^2^, *ES*	0.82 *high*	0.90 *high*	0.94 *high*	0.94 *high*	0.56 *high*	0.44 *high*	0.33 *high*
	*%_diff_,* *1-e-2*	37.62 0-0-13	40.18 0-0-13	46.90 0-0-13	17.94 0-0-13	15.28 0-1-12	11.39 2-1-10	8.53 2-3-8
Acceleration	*t* *(p)*	5.08 (<0.01)	11.44 (<0.01)	9.64 (<0.01)	9.48 (<0.01)	7.36 (<0.01)	0.97 (0.35)	0.91 (0.38)
	*η_p_*^2^, *ES*	0.68 *high*	0.92 *high*	0.89 *high*	0.88 *high*	0.82 *high*	<0.01	<0.01
	*%_diff_,* *1-e-2*	33.13 0-1-12	46.80 0-0-13	45.03 0-0-13	22.19 0-0-13	22.69 0-0-13	3.25 6-4-3	2.34 4-6-3
Deceleration	*t* *(p)*	10.04 (<0.01)	9.76 (<0.01)	10.55 (<0.01)	7.15 (<0.01)	5.31 (<0.01)	0.95 (0.36)	0.43 (0.67)
	*η_p_*^2^, *ES*	0.89 *high*	0.89 *high*	0.90 *high*	0.81 *high*	0.70 *high*	<0.01	<0.01
	*%_diff_,* *1-e-2*	52.29 0-0-13	37.15 0-0-13	35.14 0-0-13	23.44 0-0-13	26.96 0-1-12	2.95 3-9-1	1.26 4-6-3
Jump	*t* *(p)*	0.22 (0.83)	19.98 (<0.01)	14.15 (<0.01)	11.79 (<0.01)	8.48 (<0.01)	1.06 (0.31)	0.64 (0.64)
	*η_p_*^2^, *ES*	0.00	0.97 *high*	0.94 *high*	0.92 *high*	0.86 *high*	<0.01	<0.01
	*%_diff_,* *1-e-2*	1.20 1-11-1	49.99 0-0-13	53.49 0-0-13	24.64 0-0-13	23.26 0-1-12	3.49 3-5-5	1.28 4-6-3
Linear	*t* *(p)*	7.73 (<0.01)	6.08 (<0.01)	6.39 (<0.01)	5.76 (<0.01)	4.76 (<0.01)	0.41 (0.68)	0.96 (0.36)
	*η_p_*^2^, *ES*	0.83 *high*	0.76 *high*	0.77 *high*	0.73 *high*	0.65 *high*	<0.01	<0.01
	*%_diff_,* *1-e-2*	37.67 0-0-13	37.21 0-0-13	36.66 0-0-13	15.12 0-0-13	13.68 0-0-13	0.85 2-10-1	2.52 4-7-2
Small-sided game	*t* *(p)*	12.91 (<0.01)	15.06 (<0.01)	14.76 (<0.01)	23.39 (<0.01)	15.66 (<0.01)	1.12 (0.28)	2.21 (0.06)
	*η_p_*^2^, *ES*	0.93 *high*	0.95 *high*	0.95 *high*	0.98 *high*	0.95 *high*	<0.01	<0.01
	*%_diff_,* *1-e-2*	41.23 0-0-13	42.11 0-0-13	40.91 0-0-13	29.97 0-0-13	30.02 0-0-13	2.03 1-11-1	1.98 2-11-0

Note. t: t-value of repeated-measures t-test; p: significance; *η_p_*^2^: eta partial squared; ES: magnitude of effect size; %*_diff_*: percentage of differences. ^1^ Anatomical location 1; ^2^ Anatomical location 2; 1: players with higher values in anatomical location 1; e: no differences between anatomical location 1 and 2; 2: players with higher values in anatomical location 2.

## Data Availability

The data presented in this study are available on request from the corresponding author. The data are not publicly available due to the Organic Law 3/2018, of 5 December, on the Protection of Personal Data and Guarantee of Digital Rights of the Government of Spain, which requires that this information must be in custody.
